# Expression of Mipu1 in Response to Myocardial Infarction in Rats

**DOI:** 10.3390/ijms10020492

**Published:** 2009-02-03

**Authors:** Guiliang Wang, Xiaoxia Zuo, Junwen Liu, Lei Jiang, Ying Liu, Yazhu Zheng, Bin Zhang, Xianzhong Xiao

**Affiliations:** 1 Laboratory of Shock, Department of Pathophysiology, Xiangya School of Medicine, Central South University, 110 Xiangya Road, Changsha, 410008, Hunan, People’s Republic of China; 2 Department of Digestive Internal Medicine, People’ Hospital of Pingxiang City, 128 Guangchang Road, Pingxiang, 337055, Jiangxi, People’s Republic of China; 3 Department of Rheumatology and Clinical Immunology, Xiangya School of Medicine, Central South University, 110 Xiangya Road, Changsha, 410008, Hunan, People’s Republic of China

**Keywords:** Mipu1, Myocardial ischemia, Gene expression, Real-time RT-PCR

## Abstract

Myocardial ischemic preconditioning up-regulated protein 1 (Mipu1) was cloned in our laboratory. Male Wistar rats were subjected to left anterior coronary artery ligation and sham-operation and sacrificed at 1 h, 3 h, 6 h, 12 h, 24 h, 3 d or 5 d after ligation. Expression of Mipu1 mRNA and protein were assessed by Northern blotting, real-time quantitative RT-PCR, *In Situ* hybridization and Western blotting. Expression of Mipu1 was up-regulated at 3 h and lasted to 12 h with a peak at 6 h. Mipu1 mRNA and protein signals express in the endothelium and myocardium in normal and infarcted heart, mainly in infarcted zone. Fluorescent immunocytochemistry showed that Mipu1 protein was localized to the nuclei of H9c2 myogenic cells and was upregulated after the cells being exposed to H_2_O_2_. These observations indicates that Mipu1 may play a role in maintaining vascular homeostasis and protecting the myogenic cells from being injured by ischemia-reperfusion or oxidation stress.

## 1. Introduction

Acute myocardial infarction (MI) is a leading cause of death and disability in the World. Left ventricular (LV) enlargement frequently develops after MI. Myocardial loss as a consequence of infarction initiates a vicious cycle of contractile dysfunction and progressive LV dilation, referred to as ventricular remodeling. Ventricular remodeling is also affected by the infarct size, which can be limited by opening the infarct-related artery (i.e., reperfusion and arterial patency) or by the formation and development of collateral vessels [1]. The development of collateral vessels, termed angiogenesis after MI, has beneficial effects on the infarct healing, and angiogenic growth factors are considered to have roles in MI. Recently, a number of experimental studies have suggested that treatment with angiogenic growth factors can promote the development of collaterals in animal MI models [2–3], such as vascular endothelial growth factor (VEGF), an endothelial cell mitogen that is thought to function in angiogenesis [4]. During myocardial ischemia or reperfusion, many genes such as c-fos, c-jun, junB, Egr-1, HSP70 can be up-regulated [5–7], and some of these myocardial genes have been considered to be involved in the endogenous cardioprotection against myocardial ischemia-reperfusion injury. Recently, Yuan and colleagues at our lab isolated and cloned a novel gene Mipu1 (Myocardial ischemic preconditioning up-regulated protein 1, GenBank accession no. AY221750) which was characterized by a KRAB domain at the N terminal and 14 successive C_2_C_12_ type of zinc finger domains at the C terminal and up-regulated in rat heart after a transient ischemia-reperfusion procedure [8]. Jiang *et al.* further found that Mipu1 functioned as a transcription factor that bound to a specific DNA sequence TGTCTTATCGAA, with TCTTA as the core sequence and could suppress the expression of the reporter gene containing its binding sites on the promoter. Further studies showed that over-expression of Mipu1 could reduce the growth arrest induced by serum withdrawal in C_2_C_12_ myogenic cells [9]. However, the expressing pattern of Mipu1 mRNA and protein remains unclear. Accordingly, we examined the Mipu1 expression and its distribution in normal heart and experimentally induced MI heart in rats. Also, we studied sub-cellular localization analysis of the Mipu1 protein in H9c2 myogenic cells and the expression after the cells being treated with H_2_O_2._

## 2. Materials and Methods

### 2.1. Experimental Acute Myocardial Infarction

Myocardial infarction was produced by ligation of the left anterior descending coronary artery (LAD). All protocols involving experimental animals followed the local institutional guidelines for animal care, which are comparable to “Guide for the Care and Use of Laboratory Animals” published by the Institute for Laboratory Animal Research (National Institutes of Health publication No. 85–23, revised 1996). Adult male Wistar rats weighing 350 ± 500 g were anesthetized using medetomidine, 0.5 mg/kg subcutaneously (s.c.), and ketamine 70 ± 80 mg/kg intraperitoneally. The rats were connected to a respirator through a tracheotomy and the heart was rapidly exteriorised through a left thoracotomy and pericardial incision. The coronary artery was ligated about 3 mm from its origin, the heart was returned to its normal position and the thorax closed. The induction of MI was confirmed by a cardiac surface color change from reddish to a pale color and by ST-segment elevation documented by continuous electrocardiographic monitoring or the appearance of ventricular arrhythmia. Throughout the operation, the body temperature was maintained stable using a thermal plate. The anesthesia was partially antagonized with atipamezole hydrochloride 0.75 mg/kg s.c. and the rats were disconnected from the respirator. Post-operatively, rats were hydrated with 10 ml of physiological saline s.c. and given buprenorphin 0.02 mg/kg s.c. twice for analgesia. Echocardiographic studies were performed before the surgical procedure and immediately before sacrificing animals. Rats suffered ischemia for 30 min, and then post-MI 1 h, 3 h, 6 h, 12 h, 24 h, 3 d and 5 d (n=6 at each time point). The sham-operated rats underwent the same procedure, except for the ligation of the coronary artery and were used at the indicated time points to serve as a control group.

### 2.2. RNA Extraction and cDNA Synthesis

The non-infarct zone was removed from the excised heart. Infarct zone containing the infarct border zone was snap-frozen in liquid nitrogen, pulverized, and resuspended in trizole (Invitrogen, Carlsbad, CA, USA), and then RNA was extracted using guanidine isothiocyanate according to the manufacturer’s protocol. Residual DNA was removed by treatment with 5 units of DNase I (Roche Diagnostics Ltd, Lewes, UK) at 37 °C for 45 min followed by inactivation at 65 °C for 10 min. One μg of total RNA was reverse-transcribed by the reverse transcription kit (Fermentas) and PCR was performed using Cycler Apparatus (Biometra). For PCR amplification, the primers were shown in table 1.

### 2.3. Northern Blotting

The cDNA fragment corresponding to nt 120–1946 of rat Mipu1 cDNA was generated by RT-PCR. Aliquots of total RNA (20 μg) were electrophoresed and transferred to nylon membranes. After ultraviolet cross-linking, the filters were prehybridized for 1 h at 65 °C, then hybridized with α-32P-labelled cDNA probes at 65 °C for 3 h. The radiolabelled filters were then washed under stringent conditions, exposed to an imaging plate and developed using an image-analyzing system. Densities of the hybridized bands for Mipu1 were quantified densitometrically using an image-analysis program with a computer and standardized relative to the density of the glyceraldehyde-3-phosphate dehydrogenase (GAPDH).

### 2.4. Quantitative Real-Time RT-PCR

The mRNA expression of Mipu1 and GAPDH in MI was analyzed in more detail by a quantitative real-time RT-PCR method using a LightCycler rapid thermal cycler system (Roche Diagnostics Ltd.), and VEGF was also analyzed as a positive control. PCR reactions were performed in a 25 μL final volume containing 1×SYBR Premix Ex Taq (TaKaRa, Shiga, Japan), The final primer concentrations for Mipu1 and GAPDH were 10μmol/L. The amplification profiles for Mipu1 and GAPDH were 10 min at 95 °C, followed by 40 cycles of 5 s at 95 °C and 20 s at 60 °C. Each sample was analysed in triplicate with each primer set. Data were analysed using the absolute standard curve method. Standard curves were generated using a dilution series of corresponding purified PCR products. The intra- and interassay coefficients of variations were < 2% and < 3.3% respectively (data not shown). GAPDH was used for normalizing the inefficiencies in cDNA synthesis and in the amount of RNA applied. Briefly, the copy numbers for GAPDH were divided by the highest GAPDH value obtained in the experiment, resulting in a correction factor for every sample. These correction factors were then used for normalizing the absolute copy numbers of Mipu1. The normalized copy numbers were obtained by dividing the copy numbers of Mipu1 by the corresponding correction factors. For the real-time PCR analysis, the rats were sacrificed in groups experiments and in groups of the rats with sham-operated hearts at each of these times. To validate the amplification specificity of Mipu1, VEGF and GAPDH, we analyzed each PCR product by agarose gel electrophoresis before real-time detection. In addition, we analyzed the melting curve of each PCR product in each PCR session and confirmed that no non-specific products had been produced. There was rarely significant primer dimer formation during the numbers of cycles required for quantification of the PCR products from a range of experimental samples. Each RT-PCR was repeated at least 6 times to confirm reproducibility. Negative controls were checked with samples in which the RNA templates were replaced by nuclease-free water in the reactions. The PCR primers used in this study are listed in Table 1. The scores of “relative Mipu1,” which indicates the expression of Mipu1 relative to that GAPDH, were obtained by dividing the copy number of Mipu1 in a sample by the corresponding correction factor.

### 2.5. In Situ Hybridization

The myocardial tissues of 6h MI rats were used for *In Situ* hybridization. Paraffin-embedded 5-μm sections were used. The sequence of probe contain 50 bp, with the sequence 5’-caaatgtggtaaggcatatagccggagctcatctctgattcgacatcaga-3’, corresponding to nt 1871–1920 of rat Mipu1 cDNA, was used for *in situ* hybridization. Anti-sense and sense digoxigenin-UTP–labelled cRNA probes were synthesized by *in vitro* transcription with the relevant RNA polymerases (Roche Diagnostics Ltd.). Hybridization was carried out overnight at 42 °C in a humidified chamber with digoxigenin-UTP-labelled anti-sense or sense probe for Mipu1. The immunological detection of digoxigenin-labelled transcripts was performed according to the manufacturer’s protocol (Boehringer-Mannheim). Finally, the sections were lightly counterstained with Mayer’s hematoxylin and mounted with Crystal mount (Biomeda, Foster, CA, USA). Under the identical light intensity of microscope, photographs of five visual field (200×) were shot, the images were clipped at the image element of 640×480. Image Pro Plus (IPP) analysis of figures: gray scale unit was transformed to optical density unit, the yellow brown area (Mipu1 RNA staining) was chosen for spectrodensitometry. The area, mean density, and integrated optical density were measured for statistics. The inter-observer reliability and intra-observer reliability were assessed using 50 sample photographs for each grade before the grading analysis. Two investigators conducted each assessment in a blind manner; when the two investigators disagreed, an additional blind observer also made an assessment.

### 2.6. Western Blotting

We have prepared anti-Mipu1 polyclonal antibody in our previous work. Briefly, the procedure is as follows: the Mipu1 gene ORF (open reading frame) was constructed into a pQE31 vector (QIAGEN, Hilden, Germany) to express His-Mipu1 fusion protein. Then fusion protein was purified and was used as the antigen to immunize New Zealand rabbits for the generation of anti-Mipu1 polyclonal antibody. The antibody was purified by affinity purification through Pharmacia ProteinG (Pharmacia, Uppsala, Sweden) and was identified to be effectively to detect Mipu1 protein (data not shown). Proteins were extracted from the hearts using CelLytic. MT (Sigma, St. Louis, MO, USA) and were resolved on 10% SDS–PAGE and then transferred onto polyvinylidene fluoride (PVDF) membrane. The membranes were blocked overnight in phosphate-buffered saline containing 10% nonfat dry milk and 0.5% Tween-20, and incubated with anti-Mipu1 polyclonal antibody (1:200) for 2 h. Horseradish peroxidase-conjugated anti-rabbit IgG (1:1,000, Boster Biological Technology, China) was used as the secondary antibody. The immunoreactive bands were visualized using DAB (Boster Biological Technology, Wuhan, P. R. China). Mouse GAPDH (1:1,000) monoclonal antibody (Santa Cruz Biotechnology, Santa Cruz, CA, USA) was used to normalize for equal amounts of proteins and calculate the relative induction ratio.

### 2.7. Immunohistochemical Staining

The myocardial tissues of 6h MI rats were used for immunohistochemical staining. Myocardium tissues were excised carefully using microdissection tools under a stereomicroscope and then fixed in 4% paraformaldehyde in phosphate-buffered saline (PBS; pH 7.4) solution for 1 day at 4 °C, then perfused with 10% trichloroacetic acid (TCA), immersed in TCA for 1 h, washed three times with PBS, and decalcificated with 5% EDTA in PBS for at least 3 days. The tissue samples were then immersed in 30% sucrose in PBS for 1 day and embedded carefully in optimal cutting temperature (OCT) compound (Sakura Finetek, USA, Inc.). Finally, the samples were either sectioned immediately or stored at −80°C until sectioned. The serially frozen sections of tissue (10 μm) were mounted on 0.3% gelatin-coated slides. Tissue slides were fixed in cold acetone for 10 min, followed by blocking the nonspecific antibody binding in 10% normal horse serum for 40 min. The slides were then incubated with the anti-Mipu1 polyclonal antibody at a 1/50 dilution in 1% BSA in PBS at 4°C overnight, followed by three washes with PBS. The slides were further incubated with Horseradish peroxidase-conjugated anti-rabbit IgG (1:500, Boster Biological Technology, P.R. China) for 1 h at room temperature, followed by three washes with PBS. After staining, the sections were developed using a DAB substrate kit. Nuclear counterstain was performed with Gill’s hematoxylin (Thermo-Shandon, PA, USA) in all sections. Specific labeling for Mipu1 protein was seen as a brown staining in nucleus. Control sections were incubated with PBS or pre-immune rabbit serum instead of primary antibody. For each section, ten fields of view were examined. Mean density and sum area of objects, and mean density and sum area of field of view were measured by Immage-Pro plus 6.0 software. According to Shen [10], Positive Unite (PU) was calculated by the following formula:

$$\text{PU}=\frac{\left(\text{Mean Density of Objects}-\text{Mean Density of Field of View}\right)}{(1-\frac{\text{Sum Area of Objects}}{\text{Sum Area of Field of view}})\times 255}\times 100\%$$

### 2.8. Fluorescent Immunocytochemistry Study of the *S*ub-cellular Localization of the Mipu1 Protein in H9c2 Myogenic Cells and Expression after the Cell Treatment with H_2_O_2_

H9c2 myogenic cells (ATCC, Manassas, USA) were cultured on glass cover slips in a six-well plate. To study the express of Mipu1 protein after the cells being treated with H_2_O_2_, some glass covered with H9c2 myogenic cells were treated with H_2_O_2_ (0.5 mM) for 6 hours. After washing with cold PBS, cells were fixed with 2 mL of 4% paraformaldehyde for 20 min. Cells were then washed three times with cold PBS and permeabilized with 2 mL of PBS with 0.1% Triton X-100 for 15 min. After washing four times with cold PBS, cells were incubated with Mipu1 polyclonal antibody (diluted with 2% BSA, 1:50) for 2 h at 37 °C. Cells were washed three times with PBS and then incubated with the secondary antibody, CY3-conjugated goat anti-rabbit IgG (Santa Cruz) for 1 h at 37 °C. To detect the cell nucleus, specimens were further incubated with Hoechst 33258 (10 μg/mL) (Sigma) for 20 min. Background staining was removed by washing with PBS three times. The washed sections were mounted in anti-fade solution and photographed by laser scanning confocal microscopy (Nikon, Melville, NY). Images were processed with Adobe Photoshop, version 7.0 (Adobe Systems, San Jose, CA).

### 2.9. Statistical Analysis

Data are expressed as means±SEM of the indicated number of separate experiments. Statistical comparison between experimental group and control was performed using unpaired two-tailed Student’s t tests (for measurement data) or chi-square test (for percentage). *P*<0.05 was considered significant.

## 3. Results

### 3.1. Northern Blotting

**Figure 1:**
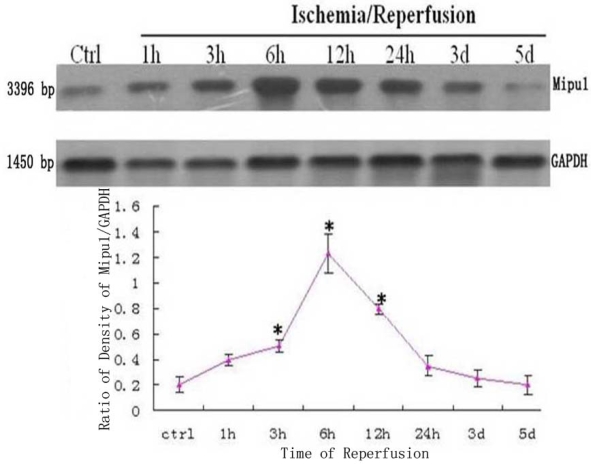
Northern blot analysis for Mipu1 mRNA in an infarct heart. GAPDH bands are shown below. * Statistically significant versus control, *P* < 0.05, n=6.

The dynamic expression of mRNA of Mipu1 in the course of MI was detected by Northern blot analysis (Figure 1). A positively hybridized band at 3396 bp was clearly observed as a single band. Mipu1 was induced to be up-regulated at 3 h after induction of MI, and achieved to the peak at 6h, and then decline, at 12 h it still express higher than control. At later stages (24 h, 3 d, 5 d after MI), Mipu1 mRNA was not strongly expressed as compared to the normal heart.

### 3.2. Quantitative Real-Time RT-PCR Analysis

**Figure 2:**
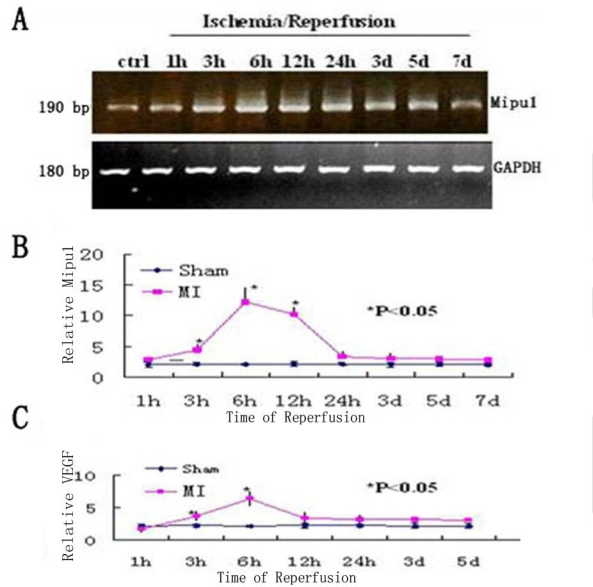
Time-dependent changes in the expression levels of Mipu1 and VEGF in the infarcted heart. A: Agarose gel electrophoresis was used to show the results of the samples at each time point analyzed by RT-PCR. B: Quantitative real-time RT-PCR analysis of Mipu1 mRNA in the infarcted hearts; C: Quantitative real-time RT-PCR of VEGF mRNA in the infarcted hearts. * Statistically significant versus sham, *P* < 0.05, n=6.

To examine the change in the level of Mipu1 mRNA in the early phase of MI, agarose gel electrophoresis was used to show the results of the samples at each time point analyzed by RT-PCR, of which GAPDH was used as the internal control (Figure 2A), then quantitative real-time RT-PCR was used to analyse the relative quantitation of the time-dependent change of Mipu1 mRNA expression in the infarcted hearts and sham operated hearts (Figure 2B). At 1 h, 3 h, 6 h, 12 h, 24 h, 3 d, 5 d after MI, the relative expression level of Mipu1 mRNA in the infarct zone was, respectively, 1.2-, 2.5- (*P* < 0.05), 6.3- (*P* < 0.05), 5.4- (*P* < 0.05), 1.9-, 1.7-, 1.5-fold greater than that in sham operated hearts. The relative level of VEGF mRNA was significantly increased at 6 h (Figure 2C), and lasted to 12 h (*P* < 0.05). The relative level of Mipu1 mRNA showed similar kinetics to that of VEGF mRNA in the infarcted rat heart. ANOVA revealed that the alterations of both Mipu1 and VEGF mRNAs after MI were significant.

### 3.3. In Situ Hybridization

**Figure 3:**
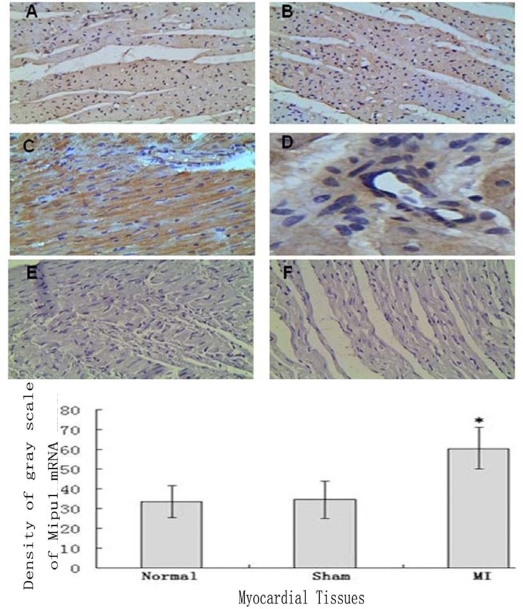
*In situ* hybridization analysis of Mipu1 mRNA in infarct endothelium and myocardium. A: Normal heart; B: Sham heart; C: Infracted myocardium; D: Blood vessels in the infracted myocardium; E: Negative control of normal heart; F: Negative control of infracted myocardium (200×). * vs Normal, *P* < 0.05, n=6.

Tissue *In Situ* Hybridization showed that not only cardiomyocytes and endothelial cells of normal heart expressed Mipu1 mRNA but also more than half of those adjacent to the infarct zone and sham-operated hearts showed weak Mipu1 protein (Figure 3A and B). The cardiomyocytes and endothelial cells in the left ventricle of 6 h MI rats expressed Mipu1 more strongly than those of sham-operated hearts (Figure 3C and 3D). Negative control of normal heart and infracted myocardium were shown in Figure 3E and 3F.

### 3.4. Western blotting

**Figure 4:**
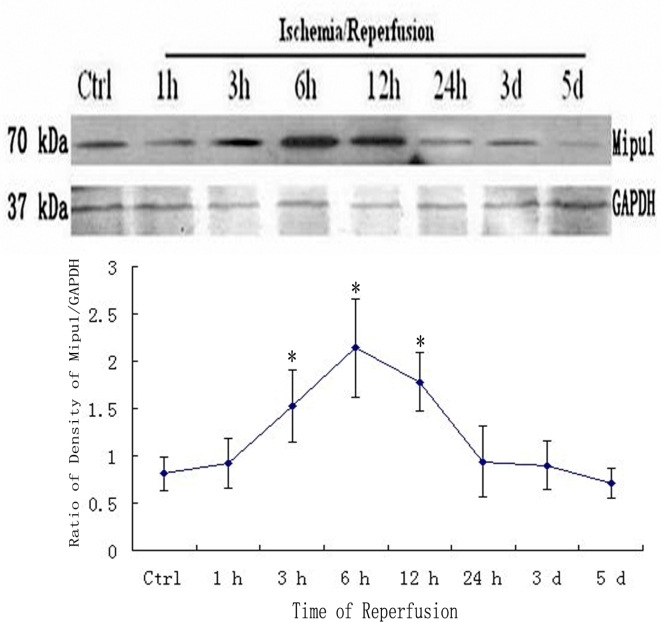
Western blot showed the expression of Mipu1 protein in the sham-operated heart and infarcted heart. The results for GAPDH are shown at the bottom. * Statistically significant versus control group, *P* < 0.05.

Western blotting analysis showed that Mipu1 protein with molecular masses of approximately 70 kDa expressed higher at 3 h after coronary artery ligation, achieved to peak at 6 h after coronary artery ligation, at 24 h it still express higher than control. At later stages (48 h, 3 d, 5 d after MI), Mipu1 protein was not strongly expressed as compared to the normal heart (Figure 4).

### 3.5. Tissue Immunohistochemistry

**Figure 5:**
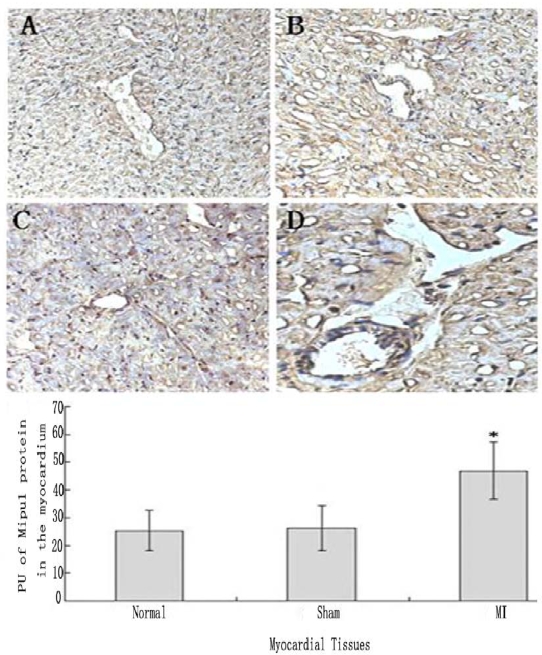
Immunohistochemical staining analysis of Mipu1 protein in cardiomyocytes and endothelial cells of sham-operated and infarct hearts. A: Cardiomyocytes and endothelial cells in normal heart; B: Cardiomyocytes and endothelial cells in sham heart; C: Infracted myocardium. D: Blood vessels in the infracted myocardium. (200×). * vs Normal, *P* < 0.05, n=6.

Tissue immunohistochemistry showed that not only cardiomyocytes and endothelial cells of normal heart expressed Mipu1, but also more than half of those adjacent to the infarct zone and sham-operated hearts showed weak Mipu1 protein (Figure 5A and B). The cardiomyocytes and endothelial cells in the left ventricle of 6 h MI rats expressed Mipu1 more strongly than those of sham-operated hearts (Figure 5C and D).

### 3.6. Fluorescent Immunocytochemistry Shows the Sub-cellular Localization of the Mipu1 Protein in H9c2 Myogenic Cells and Expression after Cell Treatment with H_2_O_2_

**Figure 6:**
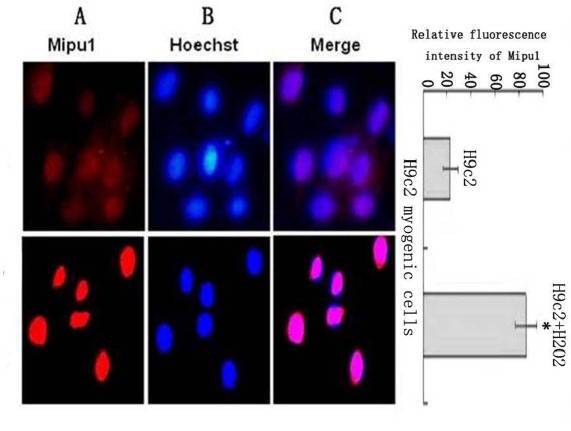
Sub-cellular localization analysis of the Mipu1 protein in H9c2 myogenic cells and the expression after the cells being treated with H_2_O_2_. The relative fluorescence intensity in the nuclear regions of multiple representative cells was determined using the Image ProPlus software and expressed as mean SEM (in arbitrary units) of six independent experiments. (A) Endogenous Mipu1 protein in H9c2 myogenic cells stained first with the anti-Mipu1 polyclonal antibody and then with CY3-conjugated goat anti-rabbit IgG (red). (B) The nuclei marked with Hoechst 33258 (blue). (C) Merged image of A and B (pink). * vs H9c2, *P* < 0.05.

Mipu1 protein was localized to the nucleus of H9c2 myogenic cells and was up-regulated after the cells being treated with H_2_O_2_ (Figure 6).

## 4. Discussion

Dion *et al*. [11] confirmed that individual zinc finger (ZF) domain recognizes DNA triplets with high specificity and affinity. By fusing ZF to repression or activation domains, genes can be selectively targeted and switched off and on. The family of Kruppel-like proteins is one of the largest families of zinc-finger proteins. These proteins contain two or more C_2_H_2_-type zinc-fingers that are separated by a conserved consensus sequence, T/SGEKPY/FX. Members of the Kruppel-like zinc finger family can function as activators or repressors of gene transcription and regulate embryonic development as well as a variety of physiological processes. Recently, studies focusing on C_2_H_2_ type zinc finger genes have suggested their unique involvement in the regulation of embryogenesis [12] and in a variety of diseases [13].

Mipu1 is a novel gene that was found to be up-regulated in rat heart after a transient myocardial ischemia-reperfusion procedure by Yuan *et al*. Further bioinformatics analysis indicated that the Mipu1 gene was composed of five exons and four introns with an open reading frame of 1827 bp and mapped to chromosome 1q12.1, encoding a 608 amino acid polypeptide with an N-terminal KRAB domain and 14 C-terminal C_2_H_2_ zinc fingers. Jiang *et al*. used a Mipu1 fusion protein GST-Mipu1, immobilized on glutathione-bound Sepharose beads to capture specific oligonucleotide sequences from a random oligonucleotide library. This studies confirmed that Mipu1 can bind specifically to the consensus sequence 5’-TGTCTTATCGAA-3’, with TCTTA as the core sequence, which suggested that Mipu1 might act as a transcriptional regulatory factor. Preliminary study in our laboratory showed that over-expression of Mipu1 in C_2_C_12_ myogenic cells were more resistant to oxidative injury when compared with the control, so it is most probably that Mipu1 is involved in endogenous cardioprotection against myocardial ischemia-reperfusion injury.

In this study, we found that Mipu1 mRNA and protein was significantly up-regulated at 3 h after MI. The present study demonstrates, for the first time, the temporal and spatial changes of Mipu1 mRNA and protein expression after myocardial infarction in rats. By using a highly sensitive real-time quantitative RT-PCR method, we found that Mipu1 mRNA achieve to peak at 6h in the infarcted area after LAD ligation. The fact that Mipu1 was up-regulated in the infarcted zone led us to propose the hypothesis that surviving ischemic cells have a special mechanism and up-regulation of Mipu1 might be related to the rate of survival of myocytes after ischemia. Mipu1 and VEGF exhibit similar temporal patterns of mRNA. Expression in the infarcted heart—another potential role of Mipu1 is the regulation of angiogenesis. Non-infarct Mipu1-positive endothelial cells may be related to the local VEGF action in the infarct remote area. In addition, our study showed the expression of Mipu1 protein in the endothelium. We found that Mipu1 immunostaining localized into the coronary artery walls throughout the myocardium and into the small arterioles of the peri-infarct and border zone areas. The mRNA and protein expression patterns show identical time-dependency, which suggests that the process of transcription and translation is very fast, once Mipu1 mRNA is up-regulated, it translates to Mipu1 protein very quickly. It was also shown that Mipu1 protein was localized to the nucleus of H9c2 myogenic cells and was up-regulated after the cells being treated with H_2_O_2_, suggesting that oxidative stress is one of important inducing factors in Mipu1 expression. These observations indicates that Mipu1 may play a role in maintaining vascular homeostasis and protecting the myogenic cells from being injured by ischemia-reperfusion or oxidation stress.

## 5. Conclusions

Our study demonstrates that the expression of Mipu1 mRNA and protein is induced to be up-regulated after myocardial infarction mainly in the infarcted area and some extent in the remote noninfarcted myocardium, suggesting that Mipu1 may be important for the survival and ultimate healing of cardiac structures after experimental myocardial infarction, which is deserved to be identified further.

## Figures and Tables

**Table 1:** The primers for the quantitative real-time RT-PCR.

Genes		Primers
Mipu1	Sence	5’-ATGCCTGCAGCCCGAGGGAAATC-3’
	Antisence	5’- CGATGATATTTGGCCTCCGGCAGGC-3’
VEGF	Sence	5’-TCTTCAAGCCATCCTGTGT-3’
	Antisence	5’-CTTTCTTTGGTCTGCATTC-3’
GAPDH	Sence	5’-AACACAGTCCATGCCATCAC-3’
	Antisence	5’-TCCACCACCCTGTTGCTGTA-3’
